# Behavioral delays in seeking care among post-acute myocardial infarction women: a qualitative study following percutaneous coronary intervention

**DOI:** 10.3389/fgwh.2025.1501237

**Published:** 2025-02-28

**Authors:** Vincenza Giordano, Caterina Mercuri, Silvio Simeone, Teresa Rea, Michele Virgolesi, Rita Nocerino, Vincenzo Bosco, Assunta Guillari

**Affiliations:** ^1^ Department of Biomedicine and Prevention, University of Rome “Tor Vergata”, Rome, Italy; ^2^ Department of Medical and Surgical Sciences, University Hospital Mater Domini, Magna Graecia University, Catanzaro, Italy; ^3^ Department of Public Health, University of Naples “Federico II”, Naples, Italy; ^4^ Department of Translational Medical Science, University of Naples Federico II, Naples, Italy

**Keywords:** women, acute myocardial infarction, ischemic heart disease, risk behaviors, delay in seeking care, social and psychosocial factors

## Abstract

**Background:**

Cardiovascular diseases (CVD) remain the leading cause of mortality worldwide, with ischemic heart disease contributing significantly to female morbidity and mortality. Despite this, women often delay seeking medical help during acute myocardial infarction (AMI), leading to poorer outcomes compared to men.

**Objective:**

To describe the early experiences of Italian women with AMI, focusing on behaviors that delay access to care.

**Methods:**

Using a phenomenological approach, in-depth interviews were conducted with 22 women hospitalized in Campania, Italy, within five days of an AMI event and their Percutaneous Coronary Intervention (PCI), to capture vivid recollections of the experience. Thematic analysis was employed to identify key themes regarding risk behaviors and delays in care.

**Results:**

Five key themes emerged: (1) vivid recollection of symptoms and experience, (2) lack of knowledge and risk perception of AMI, (3) decision-making process in seeking assistance, (4) influence of family and others on decision-making, and (5) post-AMI reflections on seeking medical care. Delays in seeking care stemmed from symptom misrecognition, social responsibilities, past healthcare experiences, and the role of family in decision-making, which either facilitated or hindered access to care.

**Conclusion:**

The findings highlight the need for targeted educational interventions that address barriers specific to women in recognizing and responding to AMI symptoms. Gender-specific training for healthcare professionals is essential to ensure timely and appropriate care for women.

## 1 Introduction

Cardiovascular diseases (CVD) are the leading cause of mortality worldwide (1). By 2030, CVDs are projected to cause approximately 23.6 million deaths globally (2). In Europe alone, CVDs account for over more than 2 million deaths among women. Among the various CVDs, ischemic heart disease (IHD) stands out as the leading cause of morbidity and mortality among women globally (3), with a prevalence of 83.6 million among women**,** an incidence of 8.67 million and a leading to 4.17 million deaths worldwide (4). In Europe, IHD accounts for 40% among women (5), while in Italy, IHD is the primary cause, representing approximately 10% of overall mortality and 28% of CVD-related deaths (6). The acute myocardial infarction (AMI) represents the most critical and severe manifestation of IHD, often requiring urgent percutaneous coronary intervention (PCI) (7). Despite women often experiencing higher early mortality rates following PCI, their long-term survival rates can be comparable to or even better than those of men when treated promptly (8–10). The VIRGO study, a large-scale analysis involving 1,465 patients across 103 centers in the United States, found that younger women report worse physical and mental outcomes, more frequent angina, and lower quality of life within 12 months following AMI compared to men (11). The literature highlights the importance of timely care (12) and indicates that delays in treatment are directly correlated with mortality (13). Women with AMI are more likely to delay seeking help compared to men (14, 15). When women recognize symptoms as abnormal, they often face challenges in obtaining timely diagnosis and appropriate treatment, a phenomenon known as the Yentl syndrome, which refers to the necessity for women to exhibit symptoms typically associated with men in order to receive appropriate diagnoses and treatments (16–18). Several factors contribute to delays in seeking care among women, including clinical factors such as different symptomatology compared to men (women often present atypical and prodromal symptoms) (19, 20), older age at the time of AMI (21), the presence of comorbidities such as hypertension, dyslipidemia, diabetes, and obesity (22) and the presence of a non-ST-segment elevation myocardial infarction (NSTEMI) or non-obstructive coronary artery disease (23), which is aggravated by limited medical knowledge about CVDs in women (24). Additionally, women, particularly younger ones, have a relatively low perception of the severity and atypical manifestations of symptoms (11) as well as minimize their symptoms and delay seeking assistance (25). Therefore, a clear and thorough understanding of symptoms can significantly reduce the number of treatments needed while improving the patient's quality of life (26, 27). Also, the psychosocial factors and socio-cultural barriers prevent timely access to care in women with AMI (28), such as the tendency of women themselves, health professionals and family members and friends to minimize symptoms, often failing to recognize their severity (28) and the cardiac nature of symptoms (29–31) when AMI does not present with the classic “Hollywood heart attack” characteristics (12). Indeed, Lichtman et al. study indicates that over half of women reported that their healthcare provider did not associate their symptoms with a cardiac problem, contributing to delays (11). Indeed, the social responsibilities such as family care and work obligations often take precedence over personal health needs (32, 33). Many women tend to procrastinate or avoid seeking help out of fear of leaving their minor children or sick relatives alone (34, 35) or to complete household chores, ensuring that nothing is left undone (36). Research indicates that women who take on the role of caregivers (37) may experience high stress during periods of intense caregiving, with negative consequences on their overall health (38–41). The lack of adequate prevention is influenced by factors such as low income, limited education, lack of control over one's life, inadequate or absent access to care, and cultural and religious influences (42). The behavioural factors, such as fear of disturbing others with symptoms and self-medication, also contribute to prehospital delay (32, 33). Despite these numerous obstacles, only a limited number of studies have explored the experiences of behaviors exhibited by women that delay access to care among those with IHD. In the study by Gallagher et al. (43), 10 women were interviewed to describe their symptomatic experiences and responses to seeking treatment for the first time for acute coronary syndrome (ACS) 3–9 months after the event, reporting that the occurrence of early warning or sometimes intermittent prodromal symptoms, the diversity of their symptom experience, the belief in low vulnerability to coronary disease by the women themselves, and the responses of healthcare providers, which did not always match their needs, contributed to rather complex and diverse decision-making processes. The study by O’Donnell et al. (44), which interviewed 42 participants (both men and women) to describe their help-seeking behavior in response to symptoms using Grounded Theory and Strauss and Corbin's (45) constant comparative analysis through semi-structured interviews, found that the mismatch between expected and experienced symptoms for participants with slowly onset AMI led to misattribution of symptoms to non-cardiac causes and prolonged delays in seeking help, whereas participants with rapidly onset AMI quickly attributed their symptoms to a cardiac cause, which accelerated appropriate help-seeking behaviors. Davis et al. (36) recruited nine women with ACS to explore how they recognized and interpreted their symptoms and decided whether to seek treatment within the context of their lives, using Grounded Theory. They discovered that all the women involved in the study delayed access to care, regardless of their ability to correctly label their symptoms. In particular, the evolving AMI group, which experienced uncertainty about bodily signals, continued to live as usual until others pushed them toward care, while the immediately recognizable AMI group quickly labeled their condition but delayed seeking care. The study by Lichtman et al. (12), which involved 30 women hospitalized with AMI to explore their experiences with prodromal symptoms and decision-making processes for seeking medical assistance, reports that participants did not accurately assess their cardiovascular risk, reported inefficient preventive health behaviors, and delayed seeking care for their symptoms. Higginson et al. (46) involved 25 women with AMI to explore the female experience of AMI, focusing on mental processes and coping strategies, using a Grounded Theory research approach with semi-structured interviews during cardiac rehabilitation programs. They found that when AMI occurs, women delay seeking help due to a lack of symptom recognition, the perception of heart disease as a “male” problem, and a preference for self-medication. Pate et al. (47) recruited 43 women diagnosed with AMI to conduct a mixed-methods assessment of the impact of ethnicity on symptom description, recognition, and treatment-seeking behavior in Hispanic and non-Hispanic women before hospitalization for AMI, using an inductive comparative approach with semi-structured interviews. They found that women and primary care physicians often underestimate prodromal symptoms of AMI and are more likely to misinterpret ischemic symptoms and delay assistance. Existing literature strongly recommends exploring the real early experience lived by women with AMI and reducing the bias of delay in conducting interviews, which could influence the vivid recollection of the lived experience (12, 36). In fact, none of these studies have thoroughly investigated the early experience, collecting data over a relatively long-time frame. Moreover, to our knowledge, no study has used a phenomenological methodology, which is ideal for understanding complex phenomena (48) and for truly understanding the early experience of Italian women with ischemic heart disease regarding the risk behaviors that delay access to care.

## 2 Aim

The study aims to describe the early lived experiences of Italian women with ischemic heart disease regarding the risk behaviors that delay access to care.

## 3 Material and methods

### 3.1 Design

The methodology used to conduct the study is hermeneutic phenomenology according to Cohen (49), which combines descriptive (Husserlian) phenomenology with interpretative (Gadamerian) phenomenology. This approach was chosen because it allows for an in-depth understanding of the experiences lived by the participants and the meanings, they attribute to these experiences (50). Cohen's phenomenological approach integrates Heidegger's thought, which is focused on the description of experiences, with that of Gadamer who emphasizes hermeneutics as the study of texts and communications, including verbal expressions and symbolic activities. This method aims to explore the nature of consciousness, focusing on the meaning of individual experiences (49). Phenomenology proves particularly effective in the analysis of complex, ambiguous and emotionally meaningful themes (51). This methodology was selected for its ability to offer a detailed and deep understanding of the experiences lived by the participants and the meanings they attribute to these experiences (51). Some authors have already successfully used this method in previous studies, for example, to explore and understand the experiences lived by women with endometriosis (52). In fact, this methodology is considered the only one that provides an authentic assessment of the experiential experience (53, 54). In order to truly explore early perception, the timing of the interviews is fundamental. Scientific literature shows that the first days of hospitalization are the ideal period to understand pre-hospitalization attitudes (55), since longer times can distort the data (43). In fact, some studies recommend analyzing pre-hospital behaviors in the early phase to obtain more accurate information (12, 36, 55). The quality of the study is guaranteed by adhering to the Consolidated Criteria for Reporting Qualitative Research (COREQ) to ensure the quality of the study (56).

### 3.2 Participants and setting

Participants were enrolled within 5 days of undergoing PCI, which followed in emergency the AMI event. This timing was chosen to balance the immediacy of emotions with the ability for critical reflection, ensuring that participants were sufficiently distanced from the acute event to reflect on their experiences, yet still close enough to provide detailed and accurate recollections. Previous studies, such as Morse (57), have highlighted that the timing of interviews can significantly impact the quality of data collected: interviews conducted too early may result in accounts heavily influenced by the immediate emotional experience, while those conducted too late may be subject to memory lapses or reinterpretations. The study was conducted in two healthcare facilities in the Campania region. A total of 22 women were enrolled through convenience sampling, selected based on their hospitalization for AMI in these facilities. All participants were admitted under emergency conditions and had undergone PCI in the preceding days. Data collection took place from October 2023 to April 2024. To be included in the study, participants had to meet the following criteria: (a) age between 18 and 85 years; (b) a confirmed diagnosis of ischemic heart disease by a cardiologist; (c) undergoing PCI; (d) physically stable and able to participate in interviews; (e) consent to be video and audio recorded during the interviews; (f) fluency in the Italian language; (g) absence of cognitive deficits.

### 3.3 Data collection and analysis

Cohen's method allows a deeper knowledge of the meaning that people give to their lived experience, and rigorous analysis is enhanced by bracketing. This initial step required interviewers to identify and suspend their personal experiences and insights that could potentially influence data analysis. Through this preliminary reflection, researchers aimed to prevent their biases from affecting the analysis (50). Cohen's method facilitates a deeper understanding of the meaning that individuals attribute to their lived experiences, and the rigor of the analysis is further enhanced by the disciplined application of bracketing, ensuring that the interpretation remains true to the participants' perspectives. This crucial step was followed by the *interviewing* phase, during which open-ended questions were employed to allow participants to freely share their experiences. The interviews were conducted in the participants' native language (Italian) by one trained researcher (V.G.). To become familiar with the methodology, two preliminary interviews were conducted, which were not included in the data analysis. Participants were asked, “*Can you describe your experience of recognizing symptoms and deciding to seek medical care during your heart attack*?” This approach prioritized participants' perspectives. During the interviews, the researchers employed a “welcome attitude”, characterized by an open and receptive approach towards the study participants (58, 59). Field notes were collected throughout the interviews. The interviews continued until no new relevant information emerged to further the study's purposes, indicating *data saturation* (60). In accordance with Strauss and Corbin's guidelines, data saturation was considered achieved when the new data collected exhibited a significant amount of repetition. Data saturation, indicated by the redundancy of emerging themes, was reached by the 22nd interview (60). All interviews, lasting between 20 and 40 min, were audio-recorded and conducted in a setting chosen by the participants. Each interview concluded when participants indicated that they had no further comments to add. After conducting the interviews, the next step involved transcribing the audio recordings verbatim, supplemented by the field notes. This transcription process was essential to accurately capture the full content of the interviews, including subtle nonverbal cues noted during the discussions (60). The transcripts were then thoroughly reviewed by each researcher, this first phase, known as immersion in the data. This phase aimed to allow researchers to deeply engage with the data, leading to the initial extraction of themes. Data analysis was conducted using a manual coding process consistent with Cohen's phenomenological methodology. This method involved iterative and in-depth readings of the transcriptions, employing line-by-line coding to identify meaningful units of data. Themes were developed progressively through an iterative process of coding, categorizing, and refining. To ensure rigor and transparency, detailed coding matrices were meticulously maintained throughout the analysis, providing a structured framework for organizing and interpreting the data. The analysis of these data was then conducted by two researchers who conducted the interviews (V.G. and A.G.), who had no prior contact with the participants, and two other members of the research team (T.R. and S.S.). Interviews were transcribed verbatim and supplemented with contact summaries. Following Cohen et al. (49), each researcher first read all the interviews to get a general sense of the content, then re-read each transcription line by line, assigning general themes to various text passages. Following transcription, the data underwent a phase of data transformation and reduction. This step involved carefully selecting relevant portions of the data and organizing them in a way that retained the essence of the participants' narratives while preparing the data for further analysis. This careful curation was essential to maintain the integrity of the data while making it manageable for the next phase of analysis. The reduced and transformed data were subjected to line-by-line coding, necessary for thematic analysis. The researchers compared the extracted themes, and no discrepancies were found between their results. Participants were informed of the identified themes in a follow-up meeting to confirm the extracted themes. All participants confirmed the themes and did not add any further insights into their experiences. This process, known as member checking, is fundamental in the phenomenology of Cohen et al. (49). As described by Cohen et al. (49) and Lincoln & Guba (61), themes should be verified with participants to ensure they accurately capture the meaning that the participants intended to convey. This process ensured the scientific rigor of the present study.

### 3.4 Rigor of the study

Adherence to Lincoln and Guba's criteria further confirmed the scientific rigor of this study. convenience sampling until saturation data ensured credibility, corresponding to internal validity and ensuring the study examined its intended focus (61–63). To further reliability of the study findings, we employed the technique of triangulation by involving multiple researchers in the data analysis process (61). This approach facilitated the generation of diverse observations and conclusions, not only reinforcing the validity of the results while enriching the analysis through varied perspectives and comprehensive understanding of the phenomenon under investigation. The criterion of confirmability was addressed by engaging participants in verifying the extracted themes, thereby ensuring that the findings accurately reflect their experiences and perceptions. Additionally, we utilized critical reflection techniques, which enabled researchers to “bracket” their own preconceptions, biases, and assumptions related to the studied phenomenon, thus minimizing potential interpretative biases and enhancing the objectivity of the analysis. Lastly, the criterion of transferability was fulfilled by providing detailed descriptions of the participants' experiences alongside their sociodemographic characteristics. This thorough contextualization allows for the assessment of the applicability and generalizability of the study results to similar settings and populations, thereby extending the relevance and impact of the research findings. Data analysis and result verification, as well as interview conduction, were performed in Italian. Translation and back-translation processes followed the World Health Organization (64) methodology for validating tools across different cultures and languages, focusing on conceptual content rather than literal equivalence. This ensured the data's meaning was preserved.

## 4 Results

Our sample consisted of 22 women diagnosed with AMI five days post-PCI, with a mean age of 64 years (SD ± 9.38; range 51–81 years). The majority had completed elementary or lower secondary education, and nearly all were either married or widowed. Most of the women were homemakers or retirees. Additionally, 91% (*n* = 20) of the participants had STEMI, while 9% (*n* = 2) had N-STEMI (Table 1).

**Table 1:** Sample's characteristics.

Code	Age	Marital status	Education	Occupation	Type of heart attack
AZF 01	81	Married	Primary school	Housewife	STEMI
BYF 02	81	Widower	Primary school	Pensioner	STEMI
CXF 03	69	Married	Primary school	Pensioner	STEMI
DWF 04	53	Married	Primary school	Housewife	N-STEMI
EVF 05	64	Married	Primary school	Housewife	STEMI
FUF 06	69	Married	High school	Pensioner	STEMI
GTF 07	51	Married	Middle school	Worker	STEMI
HSF 08	56	Divorced	Master's degree	Freelancer	STEMI
IRF 09	54	Divorced	High school	Housewife	STEMI
JQF 10	70	Widower	Primary school	Housewife	N-STEMI
KPF 11	73	Widower	Middle school	Pensioner	STEMI
LOF 12	58	Married	Primary school	Housewife	STEMI
MLF 13	72	Married	Primary school	Pensioner	STEMI
NMF 14	58	Married	Professional school	Housewife	STEMI
OLF 15	56	Married	Professional school	Employee	STEMI
PKP 16	55	Married	High school	Employee	STEMI
QJP 17	57	Married	High school	Worker	STEMI
RIP 18	72	Widower	Middle school	Freelancer	STEMI
SHP 19	73	Married	Middle school	Housewife	STEMI
TGP 20	59	Married	Primary school	Housewife	STEMI
UFP 21	67	Widower	Middle school	Housewife	STEMI
VEP 22	52	Married	Middle school	Housewife	STEMI

**Figure 1:**
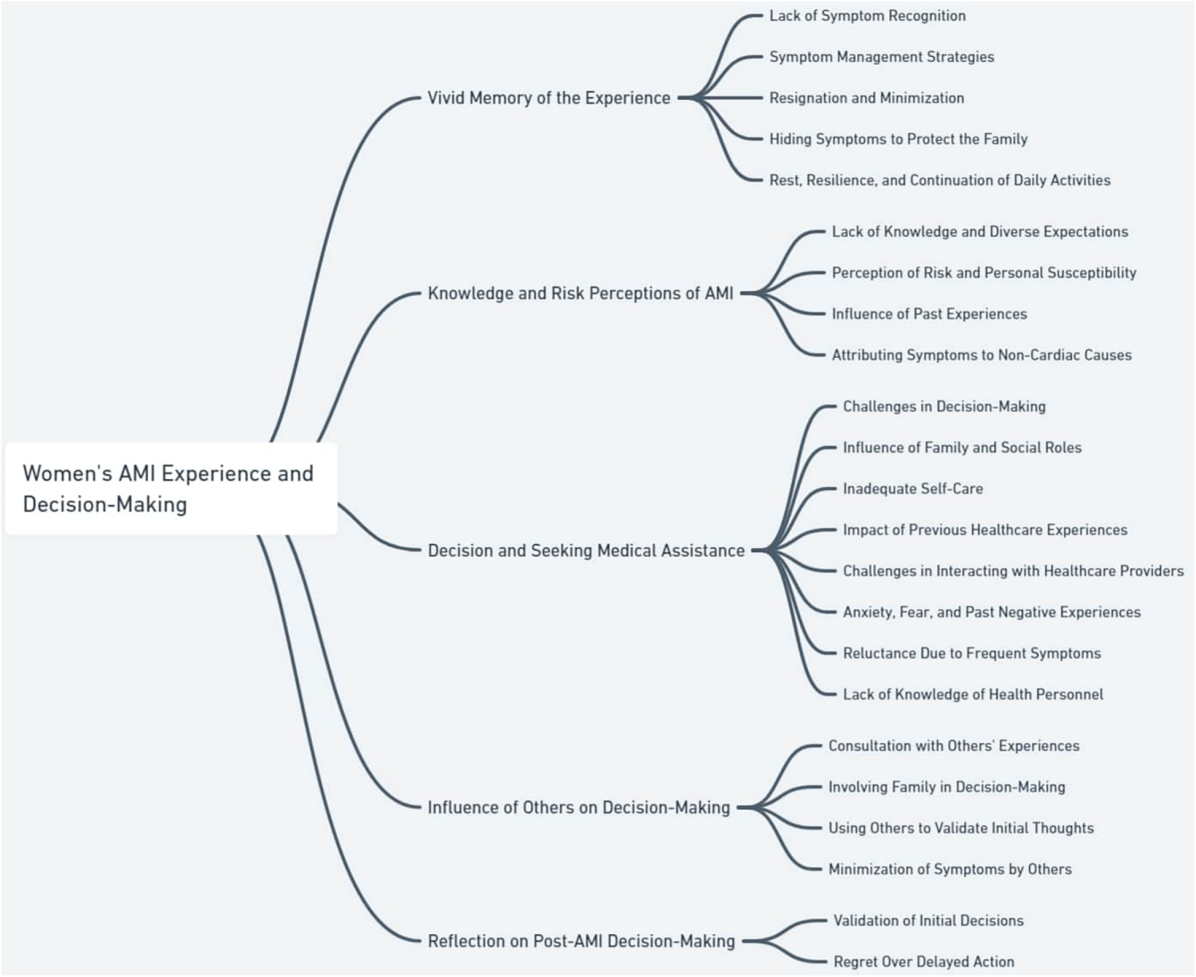
Themes and subthemes of risk behaviors leading to delays in access to services, in women with acute myocardial infarction (AMI).

Five primary themes emerged: (1) vivid recollection of the experience, (2) knowledge and perception of AMI risk, (3) decision-making and seeking assistance, (4) influence of others on the decision-making process, and (5) post-AMI reflections on seeking medical care (Figure 1 and Table 2).

**Table 2:** Theme, sub-theme and verbatim.

Theme	Sub-theme	Verbatim
Vivid memory of the experience	Lack of recognition of symptoms	“That day I had stomach pain and difficulty digesting, I confused it with acid reflux… the shoulder pain, I interpreted as cervical pain.” (HSF08) “I had these symptoms in the previous days too, but I took two or three breaths and felt fine.” (KPF11)
	Symptom management strategies	“In reality, I had high blood pressure, I put ice on my head, and it went down.” (CXF03) “I took a Maalox, then an Oki sachet… I hadn't even finished taking it when I started sweating cold.” (FUF06)
	Resignation and minimization	“When these symptoms appeared, I said “I'm going to die now, I have to accept it”.” (EVF05) “I would wake up with a bit of stomach pain and thought it was hunger… even though I had a previous heart attack, I didn't give it much thought”. (OLF15)
	Hiding symptoms to protect the family	"My son is a anaesthetist. I didn't tell my son because he does terrible shifts, and I didn't want to worry him.” (RIP18) “This chest pain started… I called my son-in-law and told him “come here, but don't scare my daughter”, because I didn't feel well”. (JQF10)
	Rest, resilience, and continuation of daily activities	"I was cleaning the floor… I lay down on the deck chair as I do every day, waiting for it to pass, but no, it kept getting worse.” (AZF01) “I was doing the kitchen, but I couldn't do the furniture and the hob properly. It started in my arm, and I sat down and took a few breaths, and it subsided for a moment. Then after a few minutes it came back and I got scared.” (KPF11)
Knowledge and risk perceptions of AMI	Lack of knowledge and diverse expectations	"I knew very little about heart attacks. I thought it would hit suddenly.” (EVF05) “No, I didn't know anything. Even though my sister had two stents, I didn't know anything; I just worried about getting regular check-ups, and they always said everything was fine.” (KPF11)
	Perception of risk and personal susceptibility	"I never thought those signs in the days before could indicate a heart attack; it never crossed my mind.” (MLF13) “I was a bit scared that something might be happening to my heart, but I didn't think it was a heart attack.” (DWF04)
	Influence of past experiences	"The pain from this heart attack was different from the one two years ago. Two years ago, the pain went away when I fell asleep.” (VEP22) “In 2019, it was a completely different situation. I barely felt a slight pain in my left arm, and now, I barely have chest pain. It was worse this time because I felt like I was dying.” (UFP21)
	Attributing symptoms to non-cardiac causes	"That day, I had this pain in my stomach and trouble digesting, so I thought it was just acid reflux.” (HSF08) “I felt a bit warm, and then I started sweating a lot, but I always thought it was just menopause because I still get my period, and I thought it was just that”. (KPK16)
Decision and seeking medical assistance	Challenges in decision-making	"At that moment, I realized something was wrong. I said, “call the ambulance because I think it's a heart attack”.” (GTF07) “The decision was mine. Everyone knows how I am—I only say “take me to the hospital” when it's serious. When the doctor mentioned the hospital, I called my husband immediately.” (QJP17)
	Influence of family and social roles	“The general practitioner was late coming, the cardiologist the same, and so my son said “enough, let's call 118.”… My son saw me, I was sweating evenly and thought to call.” (NMF14) “I have two children living with me, and I do everything at home. I think too much about them and too little about myself. That's why I didn't want to go to the hospital on the 18th-19th; I had things to do”. (GFT07)
	Inadequate self-care	"I neglected myself a bit, partly because my mother was unwell, and I had many family problems.” (VEP22)
	Impact of previous healthcare experiences	“I was reluctant to go because of my bad past experiences with hospitals, especially the one with my mother where they made us go back and forth. This experience, in particular, made me develop a strong rejection towards hospitals”. (DWF04)
	Challenges in interacting with healthcare providers	"I didn't want to go to the hospital because every time I go, they send me home saying there's nothing wrong… Even for these pains I've had… They never seem to know what I have”. (LOF12)
	Anxiety, fear, and past negative experiences	“The first time I had pain, I did not want to go to hospital, I was afraid they would hospitalise me and I would not stay in hospital” negative”. (BYF02) “After the ECG, they told me to wait for the enzyme test, but I refused. I left because the pain had subsided, but I feel much worse just being in hospitals”. (DWF04)
	Reluctance due to frequent symptoms	“I didn't go to the emergency room in the days before because I often don't feel well, and if I called an ambulance every time, it would be too much.” (FUF06)
	Lack of knowledge of health personnel	“My doctor did not understand what I had; he told me “don't worry it's a stomach ache”.” (CXF03)
Influence of others on decision-making	Consultation with others’ experiences	“My brother had different symptoms. His arm went numb.” (MLF13) “My father-in-law had a heart attack at 52 while driving and started sweating; I didn't sweat at all”. (VEP22)
	Involving family in decision-making	“I had only this symptom, then my chest, shoulder, and neck hurt, so I told my daughter because she had the same problem… and I kept telling her, “I don't feel well.”” (EVF05)
	Using others to validate initial thoughts	“I talked to my husband about these symptoms at night, and he thought the same.” (DWF04)
	Minimization of symptoms by others	“I called my neighbour, who said, “No Mrs XXXX, that's just stress from dealing with that boy all the time. Don't worry; I'll make you some chamomile tea”, and I said, “No Mrs XXXX, no chamomile, take me to the hospital, I'm not well, I'm not well”.” (KPF11) “A few days ago, I told F., “Look F., I have a strange pain in my chest”, and she said, “Mom, you know that's just your bronchitis acting up, it does that sometimes with pain here and there on your shoulders, don't worry”. We never thought it was anything like this”. (RIP18)
Reflection on post-AMI decision-making regarding seeking medical assistance	Validation of initial decisions	“Looking back, even knowing I had a heart attack, I would still not go to the hospital because I'm afraid.” (DWF04) “I would have gone privately because if you pay, everything is done properly and well”. (KPF11)
	Regret over delayed action	“If, I could go back, of course, I would go to the hospital immediately, to save myself. I wasted time.” (EVF05) “Yes, I would go straight to the hospital, immediately, because I waited 2 h.” (CXF03)

### 4.1 Vivid memory of the experience

The participants provided detailed and vivid descriptions of their experiences from the onset of symptoms to hospitalization. During the interviews, they described the behavioral strategies they employed to manage the perceived symptoms, revealing a wide range of responses that reflect a lack of awareness or understanding of the severity of their condition. NMF14 declares “I also felt them in the days before, about eight days ago. The pain wasn't so bad, though, compared to the pain that presented itself on Tuesday. Now my arms were also bothering me, but I thought it was something like that, due to the cigarette, that could cause me these complaints”.

#### 4.1.1 Lack of recognition of symptoms

Many participants initially confused their symptoms with other less serious conditions, underestimating the possibility that they might be having a heart attack. HSF08 shared, “That day I had stomach pain and difficulty digesting, I confused it with acid reflux… the shoulder pain, I interpreted as cervical pain…”. Similarly, KPF11 stated, “I had these symptoms in the previous days too, but I took two or three breaths and felt fine”. These accounts highlight how many women initially attributed their symptoms to less serious conditions, delaying their decision to seek medical help.

#### 4.1.2 Symptom management strategies

The women described a variety of behavioral strategies they employed to cope with their symptoms. Some used home remedies, like CXF03, who said: “In reality, I had high blood pressure, I put ice on my head, and it went down”. Others, like UFP21, tried to stay hydrated: “Sometimes I lost consciousness, and they told me “Drink water”, but I said “I need air””. These remedies often stemmed from attempts at self-management, which delayed medical intervention. Self-medication with over-the-counter drugs was a common practice. TGP20 stated, “I took the Oki sachet, I took 1–2, and then they told me it was a heart attack”. Some participants combined multiple medications, like FUF06, who said: “I took a Maalox, then an Oki sachet… I hadn't even finished taking it when I started sweating cold”.

#### 4.1.3 Resignation and minimization

In some cases, resignation emerged as a psychological response to the pain, with some women passively accepting the possibility of a fatal outcome. EVF05 declared, “When these symptoms appeared, I said “I'm going to die now, I have to accept it””. KPF11 added, “I wasn't worried at all because if I died, I was ready to go”. Many women minimized or ignored the symptoms altogether, hoping for spontaneous remission. For example, OLF15 recounted, “I would wake up with a bit of stomach pain and thought it was hunger… even though I had a previous heart attack, I didn't give it much thought”. This behavior reflects a combination of denial and hope that the symptoms would disappear on their own.

#### 4.1.4 Hiding symptoms to protect the family

A common tendency among the participants was to hide their symptoms from family members to avoid worrying them or out of fear that they wouldn't be taken seriously. JQF10 reported, “This chest pain started… I called my son-in-law and told him “come here, but don't scare my daughter”, because I didn't feel well”. Others, like RIP18, adds to: “My son is an anaesthetist, I didn't tell him anything because he does terrible shifts work and I didn't want to worry him”. This strategy of hiding symptoms is deeply rooted in gender roles and the social expectations that women often face as caregivers within their families.

#### 4.1.5 Rest, resilience and continuation of daily activities

Some women, hoping for a spontaneous remission of the symptoms, were convinced that by resting for a few minutes the symptoms could be alleviated, and the problem could resolve spontaneously. KPF11 reported “I was doing the kitchen, but I couldn't do the furniture and the hob properly. It started in my arm, and I sat down and took a few breaths, and it subsided for a moment. Then after a few minutes it came back and I got scared”. In contrast, other woman, despite their debilitating symptoms, showed remarkable resilience, continuing with their daily activities. AZF01 said, “I was cleaning the floor… I lay down on the deck chair as I do every day, waiting for it to pass, but no, it kept getting worse”. These accounts highlight the participants' dedication to their daily responsibilities, even at the expense of their health.

### 4.2 Knowledge and risk perceptions of AMI

The participants shared their knowledge and expectations regarding the manifestation of myocardial infarction (MI) symptoms, as well as their personal perceptions of the associated risks. These narratives reveal a nuanced understanding of how awareness and risk perception can significantly impact behavior, particularly in the context of seeking timely medical assistance. LOF12 says “I didn't understand what was happening to me; in fact, I didn't think about myocardial infarction of the heart. I just thought I was feeling sick, but I couldn't understand what it was about”.

#### 4.2.1 Lack of knowledge and diverse expectations

Many participants expressed a lack of knowledge or held misconceptions about MI symptoms. For instance, KPF11 stated, “No, I didn't know anything. Even though my sister had two stents, I didn't know anything; I just worried about getting regular check-ups, and they always said everything was fine”. This reflects a general unawareness of the severity of MI symptoms, leading to delayed recognition and response. Participants also described how their expectations, shaped by the experiences of others, influenced their understanding of MI symptoms. EVF05 mentioned, “I knew very little about heart attacks. I thought it would hit suddenly”. This expectation of a dramatic, sudden onset contributed to their difficulty in recognizing their own symptoms, which are often presented differently.

#### 4.2.2 Perception of risk and personal susceptibility

Despite having known risk factors or a family history of cardiac issues, many participants perceived themselves as being at low or no risk for MI. For example, MLF13 admitted, “I never thought those signs in the days before could indicate a heart attack; it never crossed my mind”. This perception of invulnerability, despite the presence of symptoms, further delayed their decision to seek medical care. On the other hand, participants like DWF04, who had some prior knowledge of MI symptoms, still hesitated to associate their symptoms with a heart attack. DWF04 reflected, “I was a bit scared that something might be happening to my heart, but I didn't think it was a heart attack”. This indicates a complex interplay between knowledge, personal experience, and risk perception that influences how symptoms are interpreted. Other women believed that the heart attack could not affect them as they had a strong and resilient heart due to having endured intense pain in the past. HSF08 reported “I never thought it was a heart problem because having suffered so much in life, I thought I had a very strong heart as it had been tested so many times”.

#### 4.2.3 Influence of past experiences

The participants’ past health experiences played a significant role in how they interpreted their symptoms and decided to seek care. For instance, VEP22 shared, “The pain from this heart attack was different from the one two years ago. Two years ago, the pain went away when I fell asleep, but this time, I couldn't sleep because I couldn't rest my arm”. This comparison with past experiences influenced her perception of the severity and nature of her current symptoms. Similarly, UFP21 described how the experience of a previous MI shaped her understanding of the current episode, noting, “In 2019, it was a completely different situation. I barely felt a slight pain in my left arm, and now, I barely have chest pain. It was worse this time because I felt like I was dying”. These reflections underscore the importance of considering how previous health events shape the interpretation of new symptoms.

#### 4.2.4 Attributing symptoms to non-cardiac causes

Several participants attributed their symptoms to non-cardiac causes, which further delayed their recognition of the seriousness of their condition. HSF08 explained, “That day, I had this pain in my stomach and trouble digesting, so I thought it was just acid reflux… I also had pain between my shoulder blades, but I thought it was my cervical spine acting up”. This misattribution of symptoms to other conditions highlights the complexity of symptom recognition in MI. Similarly, PKP16 mentioned, “I felt a bit warm, and then I started sweating a lot, but I always thought it was just menopause because I still get my period, and I thought it was just that”. These narratives reveal how participants' pre-existing beliefs and health knowledge influenced their interpretation of symptoms, often leading to delayed care.

### 4.3 Decision and seeking medical assistance

All participants shared their experiences and the processes that led to their decision to seek hospital care, as well as their interactions with the National Health Service (NHS). The interviews revealed that while some women were determined to go to the hospital due to the unbearable pain they were experiencing (“I couldn't wait any longer”, GTF07), others were persuaded by family members (“I didn't want to go, but F****** pulled me” and said, “Mom, stop it, you have to go!”, RIP18). These testimonies highlight the challenges faced by both patients and healthcare providers in recognizing and managing an AMI. Key issues included delayed or incorrect diagnoses, a lack of understanding of risk by emergency services, and variability in treatment received. These experiences underscore the critical importance of timely medical response and immediate support from loved ones.

#### 4.3.1 Challenges in decision-making

Several participants made the decision to seek medical help independently, often after enduring symptoms for days or even weeks, sometimes only after consulting with their general practitioner. QJP17 shared, “The decision was mine. Everyone knows how I am—I only say “take me to the hospital” when it's serious. When the doctor mentioned the hospital, I called my husband immediately”. Others, like GTF07, said, “At that moment, I realized something was wrong. I said, “call the ambulance because I think it's a heart attack”. I wanted to go to the hospital because I knew I couldn't wait any longer”.

#### 4.3.2 Influence of family and social roles

In many cases, the decision was driven by family members or friends who insisted, persuaded, or even forced them to go to the hospital. For instance, DWF04 stated, “My troponin levels were very high, and my husband took me to the hospital immediately. It was my husband who convinced me to go; I didn't want to go”. Other like, NMF14 adds “The general practitioner was late coming, the cardiologist the same, and so my son said “enough, let's call 118”… My son saw me, I was sweating evenly and thought to call”. Family responsibilities and concerns about how loved ones would react to a change in their health status or how the household would be managed in their absence also influenced the decision to seek assistance. GFT07 explained, “I have two children living with me, and I do everything at home. I think too much about them and too little about myself. That's why I didn't want to go to the hospital on the 18th-19th; I had things to do”. This reflects the common struggle of balancing self-care with caregiving responsibilities. KPF11 added “I was afraid to leave my schizophrenic son home alone… I talked to my other daughter about my symptoms, always because of the thought that if something happens to me she has to look after her brother, so not because I worried about myself. In fact, if I didn't have my son I wouldn't have worried at all”.

#### 4.3.3 Inadequate self-care

Participants acknowledged neglecting their health for years, delaying initial visits or follow-ups due to family commitments and daily pressures, which eventually impacted their health. For example, VEP22 admitted, “I neglected myself a bit, partly because my mother was unwell, and I had many family problems. I think that after my mother died, I didn't really allow myself to grieve, and that affected me”. Similarly, GTF07 mentioned, “I had been seeing a cardiologist because I've had high blood pressure since I was 18. But I wasn't taking care of myself because my mother was sick, and I took care of her for four years until she passed away”.

#### 4.3.4 Impact of previous healthcare experiences

Participants admitted that due to previous negative experiences of illness with the NHS, both personal and from loved ones, they do not access services because of a sense of mistrust. DWF04 said that “I was reluctant to go because of my bad past experiences with hospitals, especially the one with my mother where they made us go back and forth. This experience, in particular, made me develop a strong rejection towards hospitals”. This highlights how past family and personal experiences influence current illness experiences.

#### 4.3.5 Challenges in interacting with healthcare providers

Participants reported not receiving timely or thorough evaluations, nor a formal diagnosis during their interactions with the NHS. This lack of attention occurred not only in cases of atypical or less obvious symptoms but also when symptoms clearly indicated an AMI, leading to frequent misdiagnoses or unclear diagnoses. LOF12 stated, “I didn't want to go to the hospital because every time I go, they send me home saying there's nothing wrong… Even for these pains I've had… They never seem to know what I have”. This highlights the frustrations many women experience when their symptoms are dismissed or misunderstood.

#### 4.3.6 Anxiety, fear, and past negative experiences

The interviews also revealed that anxiety and fear related to medical care had a negative impact on the decision to accept or continue treatment, even when necessary. Many participants admitted to having a strong aversion to hospitals, to the point of avoiding them or refusing to continue treatment after their initial visit. DWF04 recounted, “After the ECG, they told me to wait for the enzyme test, but I refused. I left because the pain had subsided, but I feel much worse just being in hospitals”. BYF02 added “The first time I had pain, I did not want to go to hospital, I was afraid they would hospitalise me and I would not stay in hospital”. This sentiment was echoed by others who expressed similar fears and reluctance based on past negative experiences with the healthcare system.

#### 4.3.7 Reluctance due to frequent symptoms

Some women were reluctant to seek help because they frequently experienced symptoms associated with cardiac issues, leading them to perceive their situation as non-urgent. FUF06 said, “I didn't go to the emergency room in the days before because I often don't feel well, and if I called an ambulance every time, it would be too much”. This reflects a common theme where the recurrence of symptoms can desensitize individuals to the urgency of their condition.

#### 4.3.8 Lack of knowledge of health personnel

Participants reported that health personnel had difficulties recognising symptoms of cardiac origin and diagnosing AMI, complaining about the misdiagnosis and superficial attitude received. CXF03 reported “My doctor did not understand what I had; he told me “Don't worry it's a stomach ache” and so I went home to sleep”. Others, like KPF11 reported “On Monday I went to my general practioner who is also a cardiologist, and I told him about these heart problems, he told me “It's stress”. In summary, the participants” experiences reflect a complex interplay of personal, social, and systemic factors that influence their decisions to seek medical care during an AMI. These findings highlight the need for increased awareness, better communication, and more responsive healthcare systems to address the unique challenges faced by women experiencing heart attacks.

### 4.4 Influence of others on the decision-making process

All participants reported seeking advice and comparing their experiences with those of others to guide their decisions regarding the need for medical assistance. The women's choices about seeking care and managing symptoms were often influenced by their interactions and the opinions of others. LOF12 reports “I waited for my daughter for about one hour, waiting It was 4pm when the pain started, and my daughter came at 5.30 pm. I didn't want to go to hospital, she insisted”.

#### 4.4.1 Consultation with others' experiences

Many women compared their symptoms with those experienced by family members (siblings, parents) who had suffered a heart attack in the past to assess the severity of their own symptoms and decide when and if to seek help. For example, MLF13 shared, “My brother had different symptoms. His arm went numb”. Similarly, VEP22 said, “My father-in-law had a heart attack at 52 while driving and started sweating; I didn't sweat at all”. This suggests how personal or family history plays a role in shaping the perception of the current symptoms.

#### 4.4.2 Involving family in decision-making

Some women decided to inform their children as their symptoms worsened, seeking their support and advice as their health situation became more serious. Communication with family members thus played a crucial role in the decision-making process, influencing the choices regarding access to medical care. For instance, EVF05 noted, “I had only this symptom, then my chest, shoulder, and neck hurt, so I told my daughter because she had the same problem… and I kept telling her, ‘I don’t feel well’”.

#### 4.4.3 Using others to validate initial thoughts

Many women sought the opinions of others to confirm their thoughts and concerns about their symptoms and, consequently, to decide whether to seek medical assistance. However, there was also a fear of being seen as a burden or annoyance, as some people might consider these issues insignificant or even bothersome. DWF04 mentioned, “I talked to my husband about these symptoms at night, and he thought the same”.

#### 4.4.4 Minimization of symptoms by others

Conversely, some women tried to share their symptoms with others but received responses that downplayed their discomfort. For example, KPF11 recalled, “I called my neighbor, who said, “No Mrs, that's just stress from dealing with that boy all the time. Don't worry; I'll make you some chamomile tea”, and I said, “No Mrs, no chamomile, take me to the hospital, I'm not well, I'm not well”. Similarly, RIP18 said, “A few days ago, I told F., “Look F., I have a strange pain in my chest”, and she said, “Mom, you know that's just your bronchitis acting up, it does that sometimes with pain here and there on your shoulders, don't worry”. We never thought it was anything like this”. These findings illustrate the significant influence that others' experiences, opinions, and responses can have on the decision-making processes of women experiencing AMI. Whether validating their concerns or minimizing their symptoms, these interactions often play a decisive role in whether or not these women seek timely medical care.

### 4.5 Reflection on post-AMI decision-making regarding seeking medical assistance

The interviews revealed two distinct types of reflections on the decision-making process regarding seeking medical care after experiencing an AMI. JQF10 say “When something happens, a person reflects and thinks that if he had known earlier, he would have acted differently to avoid the irreparable. I should have acted immediately because I was really sick, but unfortunately, I did not realise that it was a myocardial infarction of the heart”.

#### 4.5.1 Validation of initial decisions

Some women, even after the acute event, justified their decision not to seek immediate medical assistance. They expressed a belief that, in hindsight, it might have been better to turn to private healthcare facilities, which they perceived as providing more efficient and targeted care, capable of ensuring a quicker and more appropriate response. For instance, DWF04 reflected, “Looking back, even knowing I had a heart attack, I would still not go to the hospital because I'm afraid”. KPF11 added, “I would have gone privately because if you pay, everything is done properly and well”.

#### 4.5.2 Regret over delayed action

In contrast, most study participants reported feelings of regret regarding their decision not to seek immediate assistance, acknowledging the negative consequences of their choice. For example, AZF01 stated, “Yes, I would run immediately, I wouldn't wait 15 min”. Similarly, CXF03 emphasized, “Yes, I would go straight to the hospital, immediately, because I waited 2 h.” EVF05 reflected, “If, I could go back, of course, I would go to the hospital immediately, to save myself. I wasted time, I would call the ambulance instead of going by ourselves”. HSF08 echoed this sentiment, saying, “Looking back, I would have called 118 five or six hours earlier, at 3:00 PM instead of 1:00 AM, because of the sweating, just for that”. LOF12 added, “No, no, I would go now. I think I will become like my daughter: any small issue, I'll go to a doctor”. These reflections underscore the varied responses and emotions women experience post-AMI, highlighting both the validation of initial decisions and the regret of delayed actions.

## 5 Discussion

This study aims to describe the early experiences of Italian women with ischemic heart disease regarding behaviors that delay access to care. By identifying the factors and barriers that influence decision making, it will be possible to understand the reasons for the risky behavior of women affected by AMI.

The first theme to emerge from the interviews is the “*vivid memory of experience*” of women with AMI. This theme reveals how deeply the memory of the critical event is imprinted in women's minds, significantly influencing their emotional and psychological well-being. This intense memory can also influence future decisions, such as how women respond to emergency symptoms and their trust in the health care system.

### 5.1 Lack of recognition of symptoms

The women interviewed reported that they did not recognise the symptoms as belonging to a heart problem, sometimes ignoring it or associating it with another cause. Women's lack of recognition of AMI symptoms is also reported in the literature, which not only delays access to treatment, but also leads to worse outcomes (65).

### 5.2 Symptom management strategies

Women AMI clearly described the behavioral strategies they employed to manage the perceived symptoms. The study shows that some women tend to underestimate AMI symptoms, resorting to over-the-counter medications such as anti-inflammatories and analgesics, and dismissing the possibility of a heart attack due to a lack of knowledge about the symptoms. This practice can be risky and exacerbate the condition (66). The literature supports that many women prefer self-treatment to avoid disturbing others and to maintain control over their symptoms, leading to delayed medical assistance for 92% of women, as reported in Higginson's study (46). In developing countries, easy access to over the counter and prescription medications contributes to self-medication among women, who tend to manage various short-term ailments autonomously (67, 68). In contrast, women when faced with unclear symptoms, tend to adopt generic coping responses such as moving around, resting, or drinking something, as also demonstrated in studies by Meiscke (69) and Dempsey et al. (70).

### 5.3 Resignation and minimization

The women often reported feelings of resignation and interpreted the symptoms as signs of an inevitable fatal outcome, which reduced their motivation to seek medical help. This sense of helplessness may also be influenced by personal religious beliefs (71). Women, also, tended to downplay their symptoms, sometimes reducing their severity or stating that they were not concerned about the possibility of death; an attitude of minimising symptoms also discussed in the study by Rosendelf et al. (25), while continuing to carry out household work and activities of daily living.

### 5.4 Hiding symptoms to protect the family

Coping strategies identified in the study include ignoring symptoms, hiding them from others, and not recognizing them as signs of a heart attack. These behaviors share the denial mechanism, a common self-defense strategy that leads to rejecting ideas associated with unpleasant experiences (72). Denial is frequent among patients with signs of AMI (73, 74) and can cause significant pre-hospital delays (75–77), as demonstrated by O'Carroll et al. (78), where AMI patients who waited more than 4 h before seeking medical assistance had significantly higher denial scores. Moreover, the denial defense mechanism can persist over time, preventing patients from having a clear view of their condition, avoiding medical recommendations, and inadequately managing disease-related problems (79). Women with mild or intermittent symptoms often ignore the initial signs, hoping that they will resolve themselves (69) or consider them to be minor problems (79) or to avoid being a burden on others, and to avoid causing concern and disturbance to doctors and family members (46, 80, 81) and to avoid these they may minimize, delaying help-seeking even in the presence of intense pain, especially at night or on weekends (25).

### 5.5 Rest, resilience and continuation of daily activities

During the onset of symptoms, some enrolled women choose to rest, hoping that the symptoms will improve, while others continue to manage their daily responsibilities, demonstrating strong dedication to their family and professional duties, behaviour also reported by other studies (36, 81). Turris demonstrates that women's adherence to daily commitments is deeply connected to their sense of personal identity (81), while Davis et al. add that during an acute event, women try to ensure that nothing is left unfinished, attitude also carried out by female workers, who try to obtain leave or find a replacement before going to the emergency room to ensure that their responsibilities are managed by capable people (36).

The second theme concerns the analysis of “*knowledge and c risk perception of AMI*”. The lack of knowledge about AMI symptoms is a global issue: while chest pain is recognized as a primary symptom (82, 83), other less known symptoms are not recognized (84). The poor awareness of atypical AMI symptoms indeed contributes to delays in seeking help and to negative health outcomes (26, 68, 82, 85–87).

### 5.6 Lack of knowledge and diverse expectations

Many study participants reported not having sufficient information about AMI symptoms, and even among those who had some knowledge, it was often limited and/or superficial. As reported by Gkika et al. (88), knowledge of symptoms is mostly obtained through experiences of family members or friends rather than through reliable medical sources. It is important to note that although AMI patients tend to have inadequate knowledge compared to patients with other heart problems, their level of awareness is still higher than the average (88). In fact, an Italian survey conducted by Maffei et al. (89) showed that 69.8% of Italian women are aware of cardiovascular diseases as the leading cause of death. These data are significantly higher than the awareness percentages of women in the United States (56%) according to Mosca (90), and women in Australia (32%) according to Hoare et al. (91). However, mere knowledge of symptoms is not sufficient to correctly attribute AMI symptoms (92), as emotional factors also play a significant role during an acute event (93, 94). The study by Albarqouni et al. showed that STEMI patients with a history of AMI or stent placement had significantly lower knowledge levels compared to those without such experiences (95). Moreover, Strömbäck et al. (96) found that the presence of atypical symptoms during a second AMI does not predict a longer delay in seeking assistance. This observation may be due to the fact that the history of AMI is associated with an increase in symptom congruence (96). However, the study by MacInnes suggests opposite behavior, where women with a previous history of AMI seemed more likely to seek immediate medical assistance (27). Some participants had difficulty recognizing AMI symptoms because they did not match their expectations due to the influence of descriptions of heart attack symptoms, such as severe chest pain, reported by family members. Indeed, the literature points to the importance of the correspondence between symptoms and expectations in the recognition of AMI (97–101), as stated by Fox-Wasylyshyn et al., who report that “the extent to which personal experience with symptoms of AMI aligns with expectations of an AMI” is crucial (98).

### 5.7 Perception of risk and personal susceptibility

The study participants, particularly those with known risk factors and typical AMI symptoms, underestimated their cardiovascular risk; this lack of awareness is particularly evident among those with previous heart attacks or a family history of heart disease. Maffei et al. in fact report how this misunderstanding is particularly evident among younger women, who may not pay sufficient attention to cardiovascular health due to a lack of experience and knowledge (89). Dempsey et al. have also demonstrated that many women are skeptical about their cardiovascular risk due to erroneous beliefs and the desire to maintain control over their experience, associating such pathologies only with the male gender (70). Some women are also affected by an “illusion of invulnerability” (102), believing they are protected due to a healthy lifestyle and/or the absence of a family history of heart disease (102–104).

### 5.8 Influence of past experiences

The enrolled women compare their own symptoms with those of family members or friends with a previous AMI, considering their symptoms severe only when they were similar to those already known (73). Petrie and Weinman, indeed support that the personal experience of heart disease through a family member tends to foster the perception of a serious illness and can contribute to reducing treatment delays (105). However, the data suggests that this influence may be more closely related to the degree of similarity or difference in symptoms rather than the experience itself (105). Medical and nursing staff play a crucial role in educating women with AMI about knowledge regarding the onset, characteristics, and progression of AMI, risk factors, and preventive measures (88). Nurses should be able to correct patients' misconceptions about their condition and educate them about symptoms and risk behaviors (106).

### 5.9 Attributing symptoms to non-cardiac causes

The women involved in the study, past experiences also influence the perception of symptoms and the decision to seek help: women with previous heart attacks often minimize new symptoms, attributing them to alternative causes such as allergies or indigestion, as reported in numerous studies (25, 27, 106, 106).

The third theme examines women's “*decisions and seeking medical assistance*”, to explore the challenges they face in the decision-making process. Difficulties include the influence of family and social roles, which can both support and hinder the decision to seek help, an inadequate self-care, influenced by previous negative experiences with health services and anxiety and fear arising from past experiences. Further challenges arise from reluctance to seek care due to the frequency of symptoms and lack of knowledge about health professionals. These factors combined can complicate interaction with care providers, negatively affecting the timeliness and effectiveness of seeking medical care.

### 5.10 Challenges in decision-making

The enrolled women delayed seeking medical assistance until the symptoms became unbearable, as reported in two studies (73, 107). Despite the autonomous decision of the enrolled women to seek assistance, however, instead of going directly to the Emergency Room, many women chose to contact their general practitioner or a private cardiologist, prolonging the time to intervention, behaviors reported also in studies by Gallagher et al. (43), Johansson et al. (107), Coventry et al. (108), and McKee et al. (109).

### 5.11 Influence of family and social roles

The interviews reveal that in many cases, family members convinced or forced the women to seek medical assistance, indicating a lack of self-efficacy or awareness (73). Family responsibilities significantly influenced the decision to seek medical assistance: women, in fact, procrastinate or avoid seeking help for fear of leaving disabled or elderly family members without adequate care (34, 35). The cause may be found in cultural and social norms, which often attribute the primary role of family care to women, leading them to neglect their own health (81, 110, 111).

### 5.12 Inadequate self-care

Women neglect their own health due to family commitments and the emotional burden associated with caring for sick or elderly family members, resulting in delays in check-ups and the management of medical conditions (34, 112). This role as a caregiver may make them less inclined to seek urgent medical assistance, as indicated by Turris et al. (111), reducing attention to their own health concerns (29, 113).

### 5.13 Impact of previous healthcare experiences

The women interviewed frequently reported that the healthcare system offers insufficient responses, both in emergency situations and during visits with general practitioners. This often leads them to consult their family doctor before going to the emergency room, as was also highlighted by a Swedish study (102).

### 5.14 Challengers in interacting with healthcare providers

The women interviewed often reported a lack of trust in healthcare professionals, as they tended to play down symptoms, attribute them to non-cardiac causes or send patient's home. This mistrust, also reported in the literature, is further accentuated when a clear diagnosis cannot be obtained, mainly due to difficulties in describing symptoms, making the diagnostic process complex and ambiguous (114). For example, in the study by Gallagher et al. (43), GPs experienced the same difficulties as patients in recognizing a cardiac cause in female symptoms, despite the fact that these could signal potentially serious events.

### 5.15 Anxiety, fear and past negative experience

The interviews also reveal that anxiety and fear related to medical care are significant barriers. Many women avoid treatments due to past negative experiences or generalized anxiety about healthcare facilities (114), compounded by negative attitudes from healthcare providers and access issues (114).The enrolled women, also report that past negative experiences, both personal and family-related, and distrust in the healthcare system, fueled by inadequate treatments or previous medical errors, lead them to avoid assistance even in the presence of severe symptoms. Hadid et al. (115) found in 32 AMI patients a lack of trust in hospitals or medical personnel due to previous negative experiences with close relatives, and they fear dying due to medical negligence or incompetence.

### 5.16 Reluctance Due to frequent symptoms

The women participating in the study reported that they procrastinated seeking medical help for fear of appearing to seek help too often (“I often don't feel well and if I had to call for help every time it would be too much”). The women participating in the study reported that they procrastinated seeking medical help for fear of appearing and being judged as those who ask for help too often, due to the frequency with which they manifest symptoms, a concern also reported in the study by Hadid et al. (115),. This behaviour suggests a kind of self-censorship due to a fear of social judgement and a concern that they do not want to “disturb” the healthcare system, even when their symptoms are serious.

### 5.17 Lack of knowledge of health personnel

Interviews reveal a difficulty in recognizing cardiac symptoms and ineffectiveness of responses from healthcare personnel, as reported in numerous studies (116, 117), where severe symptoms are misinterpreted as less severe symptoms (bronchitis or stress), leading to inadequate treatment (118). Rosenfeld et al. report the case of a woman with chest pain who was diagnosed with a gastrointestinal problem (74). Sjostrom-Strand & Fridlund instead, describe the case of a 39-year-old patient who was reassured by the doctor that she was too young to have a heart attack, showing how age-based biases can lead to misdiagnoses and treatment delays (35). Furthermore, among the women interviewed, many had been diagnosed with bronchitis and/or stomach ache, similar to those reported in the study by Berg Gundersen et al. (119). This attitude of minimising gravity is consistent with Healy's (16) “Yentl syndrome”, in which women's heart problems are not recognised by health professionals until they reach maximum severity.

The fourth theme analyzes how the “*influence of others on decision-making*”. The influence of others, in fact, can act as a catalyst or a brake in the decision to seek timely medical attention.

### 5.18 Consultation with other's experience

The interviews showed that if the symptoms experienced by the women do not correspond to those already known or experienced, for example, by family members, the women are less likely to recognise them as signs of AMI. In fact, according to the study by Davis et al. when women fail to interpret their symptoms correctly, they often consult other people; some do so immediately, others later in the course of the symptoms (36). This comparison serves to obtain advice and confirmation of their suspicions, even among women who have already had a heart attack, due to the atypical nature of the symptoms, which do not correspond to those commonly known (36).

### 5.19 Involving family in decision-making

Women interviewed, report that family play a significant role both as secondary decision-makers and as emotional support: their presence can incentivise seeking care, suggesting that the decision to seek care is often influenced by social pressure and perceived support (120). Sharing symptoms with loved ones may help patients overcome fear or denial of the illness, reducing the delay in accessing pre-hospital care (29, 93, 97, 102, 107, 121–126). Alonzo's study (127) found that, among more than 1,000 patients with AMI, 93% consulted family members or friends before seeking professional help, with both positive and negative effects, sometimes waiting for family members to return home to discuss symptoms (111, 128, 129). This behaviour could be explained to the lack of autonomy of some women, who depend on men to make decisions, asking men's permission before spending money on medical care (129, 130).

Many women report a sense of relief when the decision to go to hospital is made by a family member, and they often prefer to wait until the most convenient time for their partner, especially if their symptoms are stable, rather than calling the emergency room immediately (36). However, the delay time tends to increase if the patient consults a spouse or another family member, compared to when a third-party present during the acute event immediately makes the decision to seek medical assistance (125). In addition, the presence of a third party, during the onset of the acute event, has been shown to be influential in the decision to seek medical help (126, 131–133).

### 5.20 Using others to validate initial thoughts

In order to confirm their initial perception of the symptoms, many women interviewed chose to share them with friends or family members, seeking a comparison to validate their feelings. This sharing process is a crucial step in strengthening their awareness of the severity of the situation. When women discuss their symptoms with family members and receive confirmation that these could be related to a heart attack, their decision to seek medical attention becomes more solid and urgent (36), which allows them to overcome doubt or denial and act more confidently and quickly.

### 5.21 Minimizations of symptoms by others

Conversely, some women reported that their concerns were minimized by family and friends, who attributed the symptoms to less serious causes such as stress or fatigue, often accompanied by non-medical suggestions such as taking chamomile or eating biscuits, leading to an underestimation of the problem. Therefore, if sometimes family and friends can validate symptoms and encourage help-seeking (134, 135), at other times they may minimize symptoms to reassure, hindering care (112, 136).

The fifth theme includes “*reflection on the post-AMI decision-making*” process regarding seeking medical assistance in the presence of symptoms. Many women, after experiencing an AMI, rethink the decisions they made during that critical event, with two main feelings: validation of initial choices and regret for delaying action. Validation of initial decisions occurs when patients feel they acted in the best possible way, considering the information and emotions available to them at the time. This reflection process leads them to justify their choices, especially if the severity of the symptoms was not obvious or if family support was relied upon to make the decision. On the other hand, however, many express regret for having delayed in seeking medical help. This regret stems from the knowledge that earlier action could have reduced the risks and improved the outcome of their condition. This process of reflection, between validation and regret, provides a valuable opportunity to raise awareness and improve the response to cardiac emergencies in women.

### 5.22 Validation of initial decisions

The interviews show that some women, despite having experienced heart attack symptoms, would have chosen not to seek immediate medical assistance due to fear or distrust in the healthcare system. As demonstrated in Hadid's et al., study (115). Participants fear that negligence or poor medical decisions, such as those that have already affected some of their family members, could put their lives at risk. The interviews also reveal that choosing private facilities can ensure faster and higher quality care: in fact, some women believe that paying for a medical service corresponds to better quality care, in line with the study by Owusu Kwateng et al. (137).

### 5.23 Regret over delayed action

Others, however, reflecting on these experiences, expressed regret for not acting promptly. Statements like “I would go right away” and “I would have called 118 sooner” highlight the recognition of the importance of a quick response in the event of a cardiac emergency. After overcoming the critical phase, women develop greater awareness of the risks and urgency of heart attack symptoms of AMI, positively influencing their future healthcare decisions (14). Adopting a quicker response in the future may be influenced by this belated recognition of urgency (138).

The adoption of a qualitative approach to explore the meaning of AMI for women is likely to have led to the creation of a more detailed picture that goes beyond pre-existing models and theories. Consequently, this study represents an important opportunity to gain a deeper understanding of the lived experiences of women with AMI. The multiple themes and sub-themes that emerged from the interviews enriched this understanding and can serve as a basis for the design of targeted and specific educational programmes.

## 6 Limitation

This study has a limitation. The samples consists of women with AMI from a single Southern Italy (Campania), which may not adequately reflect social and cultural variations across other Italian regions or countries. Despite this limitation, the study's focus on the lived experiences of women with AMI who delayed accessing healthcare services highlights an important effort to explore this issue.

## 7 Implication

However, our study has several important implications. Our findings could be useful for improving the management of heart attacks and reducing treatment delays by enhancing education and awareness of symptoms and their severity through targeted programs and the development of tailored interventions for this specific population. These should help women recognize warning signs promptly and seek immediate assistance. Promoting self-recognition of symptoms, including less-known ones, and raising public awareness about the variability of heart attack symptoms can improve timely responses. It is crucial to address psychological and social barriers, such as the fear of being a burden, and consider the influence of family roles on symptom management behavior. Moreover, awareness campaigns and ongoing training for woman and healthcare professionals are essential. Healthcare policies should integrate support for caregivers and develop specific educational strategies. Future research should also consider the impact of women's risk behaviors on quality of life.

## 8 Conclusion

This phenomenological study identified five key themes: vivid memory of the lived experience, knowledge and perception of AMI risk, decision and request for assistance, the influence of others in decision-making, and reflection on post-AMI decision-making. These themes encompass the challenges women face, including recognizing and managing symptoms, misconceptions about AMI risk, reliance on others for decision-making, and the emotional impact of delayed medical assistance.

The findings offer important insights into the risky behaviours women may exhibit during a heart attack and highlight the critical role of nurses and healthcare professionals in prevention and care. Through tailored education and efforts to raise awareness of AMI symptoms, healthcare providers can help women to recognise early warning signs, reduce hesitation and take prompt action. Nurses are in a unique position to build trust, allay fears and empower patients to prioritise their health, thereby improving outcomes for this vulnerable group. By addressing challenges such as fear, anxiety and limited knowledge, nurses can encourage patients to overcome uncertainty and make confident health decisions. This comprehensive approach not only improves early detection and management of AMI, but also strengthens preventive measures, ultimately promoting better long-term health outcomes for this vulnerable population.

## Data Availability

The raw data supporting the conclusions of this article will be made available by the authors, without undue reservation.
